# Socioeconomic Diversity in Admissions to MD-PhD Programs, 2014-2019

**DOI:** 10.1001/jamanetworkopen.2024.1951

**Published:** 2024-03-12

**Authors:** Mytien Nguyen, Jose E. Cavazos, Shruthi Venkataraman, Tonya L. Fancher, Sarwat I. Chaudhry, Mayur M. Desai, Dowin Boatright

**Affiliations:** 1 Department of Immunobiology, Yale School of Medicine, New Haven, Connecticut; 2 South Texas MSTP, University of Texas Health San Antonio, San Antonio; 3 Department of Emergency Medicine, New York University Grossman School of Medicine, New York; 4 Division of General Internal Medicine, University of California, Davis, School of Medicine, Sacramento; 5 Department of Medicine, Yale School of Medicine, New Haven, Connecticut; 6 Department of Chronic Disease Epidemiology, Yale School of Public Health, New Haven, Connecticut

## Abstract

This cohort study of applicants to US MD-PhD programs examines the association of application outcomes with family income.

## Introduction

Physician-scientists play a unique role in translating research to clinical practice. Diversity among physician-scientists is essential for biomedical innovation and equitable health care.^1^ MD-PhD training programs represent a critical pathway for the development of physician-scientists. Although first-generation college students have been less likely to be accepted into MD-PhD programs,^2^ little is known about how application and acceptance rates vary across household income. This study aims to examine trends in application and acceptance to MD-PhD program by family income.

## Methods

We conducted a retrospective cohort study of applicants to US MD-PhD programs between 2014 and 2019 using deidentified data from the Association of American Medical Colleges. Data and descriptive statistics are described in Supplement 1. We estimated the relative risk of acceptance to at least 1 MD-PhD program across income using modified Poisson regression with robust error variance, adjusting for students’ self-reported race, ethnicity, sex, grade point average, Medical College Admission Test results, number of publications, and total MD-PhD programs applications sent. Statistical significance was 2-sided with *P* < .05 indicating statistical significance, and analyses were performed in July 2023 using Stata version 18.0 (StataCorp LLC). We followed the Strengthening the Reporting of Observational Studies in Epidemiology (STROBE) reporting guideline. This study was deemed exempt by the Yale institutional review board, and informed consent requirements were waived because data were deidentified.

## Results

**Figure 1.**
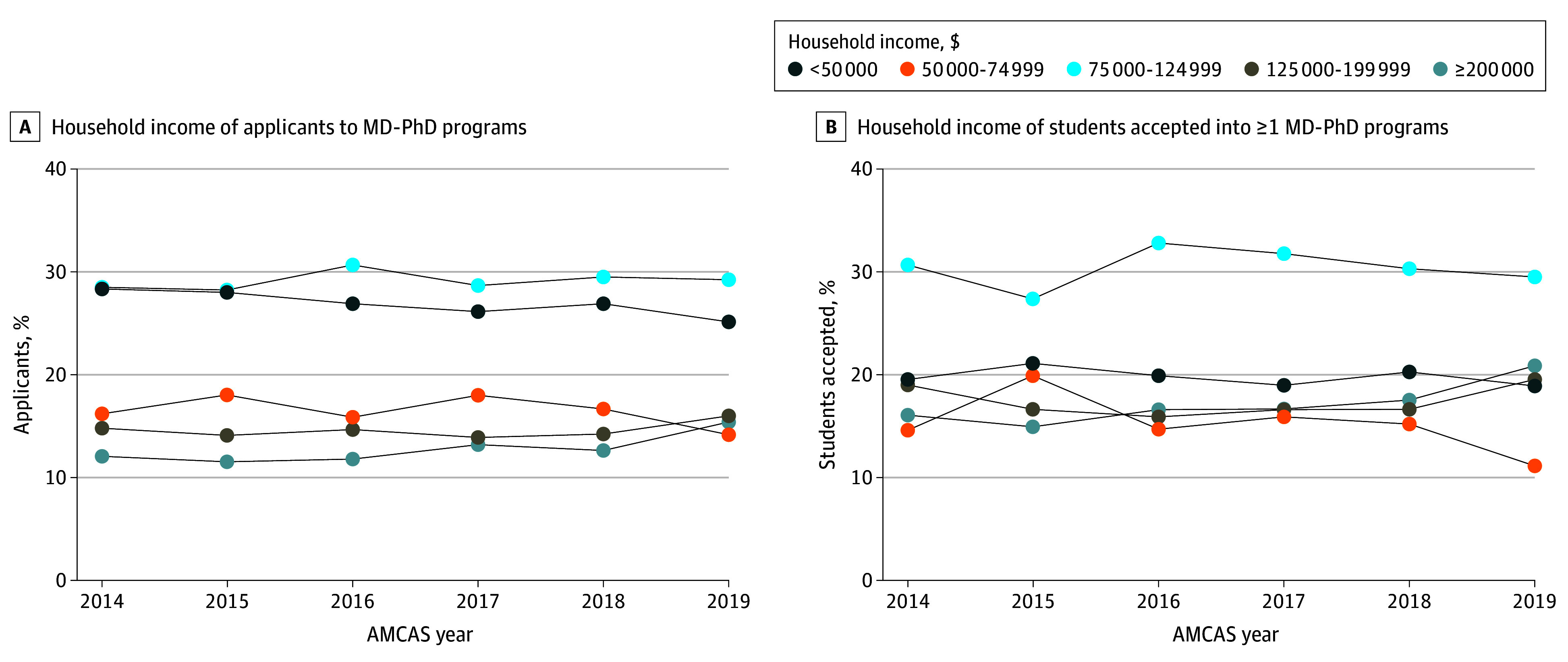
Childhood Household Income Profile of Students Who Applied and Were Accepted Into MD-PhD Programs AMCAS indicates American Medical College Application Service.

Between 2014 and 2019, 10 953 students applied to MD-PhD programs. Of these, 4724 students (43.1%) were female; 5020 (52.1%) were White, 2243 (23.3%) Asian, and 805 (8.4%) multiracial. Among MD-PhD applicants, 4959 (45.3%) were accepted into 1 or more MD-PhD program. The percentage of applicants reporting household income less than $50 000 decreased annually, from 28.36% in 2014 to 25.14% in 2019 (annual percent change [APC], −0.57%; 95% CI, −0.94% to −0.21%; *P* = .01) (Figure 1). No significant change was found for other income categories. In contrast, the percentage of accepted students reporting household income greater than $200 000 increased annually, from 16.10% in 2014 to 20.87% in 2019 (APC, 0.90%; 95% CI, 0.08% to 1.72%; *P* = .03), with no significant change for other income categories.

**Figure 2.**
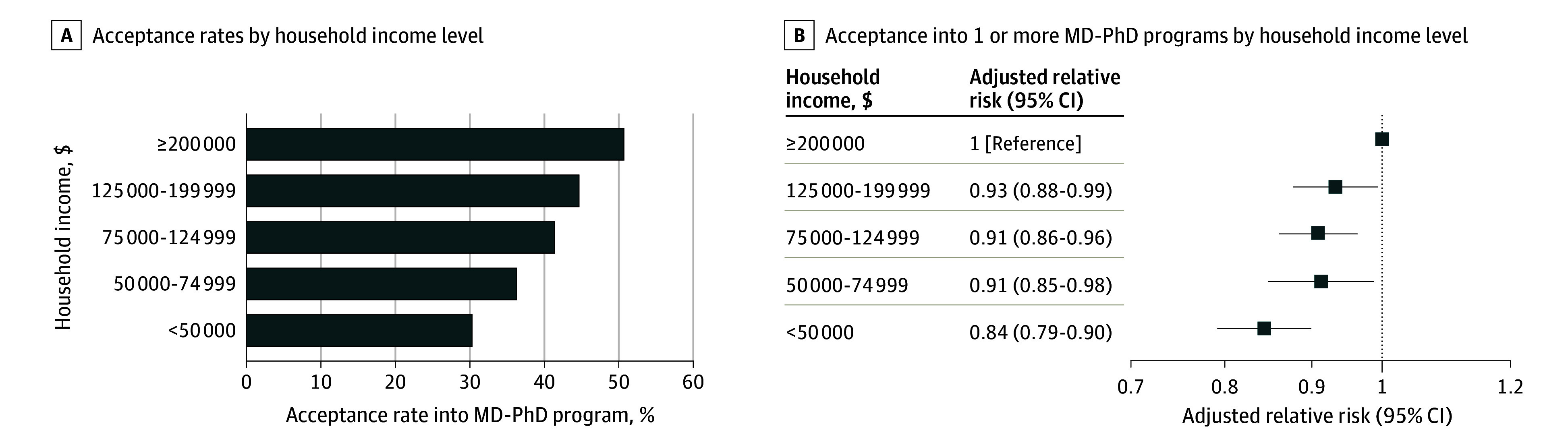
Admission Outcomes for MD-PhD Applicants by Household Income In panel A, rates of acceptance into MD-PhD program by childhood household income; B, relative risk of acceptance into 1 or more MD-PhD program for MD-PhD applicants adjusted for number of MD-PhD applications submitted, number of publications, grade point average, Medical College Admission Test quartile, sex, race, and ethnicity.

Combining applicants across all years, while 50.3% of applicants from household income greater than $200 000 were accepted to MD-PhD programs, only 29.9% of applicants from household income less than $50 000 were accepted (Figure 2). In the fully adjusted model, applicants from household income less than $50 000 were 16% less likely than their peers to be accepted into an MD-PhD program (adjusted relative risk, 0.84; 95% CI, 0.79-0.90).

## Discussion

In this study, MD-PhD applicants from household income less than $50 000 had a 16% lower acceptance rate than their wealthier peers between 2014 and 2019. Our study advances our understanding of the influence of class on MD-PhD application and matriculation. The step-wise association between students’ income and MD-PhD program application and acceptance rate is consistent with prior research demonstrating the association between income and MD programs admission,^3^ suggesting pervasive socioeconomic inequity in access to the medical profession.

The decline in low-income applicants is noteworthy and may be due to the significant financial investment in pre–MD-PhD preparation, including research years and the cost of the increasing time to independent physician-scientist career.^4^ A key finding from our study is that even after accounting for applicants’ tests scores and prior publications, low-income applicants remained less likely than their affluent peers to be accepted into an MD-PhD program. These findings suggest that MD-PhD admission committees may select applicants based on characteristics more associated with privilege than merit, which may include undergraduate institution prestige,^5^ exposure to a high-impact mentor,^6^ and having a parent with a doctoral degree.

Study limitations include that childhood income does not necessarily reflect wealth and opportunity due to geography and other unmeasured factors. Results therefore may underestimate the degree of socioeconomic inequity. Our analysis did not include MD-PhD program level characteristics, and it is unclear how characteristics such as program prestige, MD-PhD program Medical Scientist Training Program status, and overall medical school National Institutes of Health funding may influence equity in admissions.

Diversity in the physician-scientist workforce is critical for innovation in the biomedical sciences and patient care. To promote socioeconomic equity in MD-PhD program admission, program directors and admissions officers should rely on holistic measures such as grit, resilience, and distance traveled, in addition to traditional academic metrics.

## Notes

Supplement 1.
eMethods.

Supplement 2.
Data Sharing Statement
