# Supplementary material for: Socioeconomic Diversity in Admissions to MD-PhD Programs, 2014-2019
Source: JAMA Netw Open. 2024 Mar 12;7(3):e241951. doi: 10.1001/jamanetworkopen.2024.1951 (PMC10936114; doi:10.1001/jamanetworkopen.2024.1951)
Supplement: Supplement 1. — eMethods. [file jamanetwopen-e241951-s001.pdf]

Supplemental Online Content

Nguyen M, Cavazos J, Venkataraman S, et al. Socioeconomic diversity in admissions to MD-PhD Programs, 2014-2019. *JAMA Netw Open.* 2024;7(3):e241951. doi:10.1001/jamanetworkopen.2024.1951

eMethods.

This supplemental material has been provided by the authors to give readers additional information about their work.

© 2024 Nguyen M et al. *JAMA Network Open.*

Supplemental Methods

On the American Medical College Application Service (AMCAS), students self-reported race and ethnicity, sex, grade point average (GPA), and Medical College Admission Test (MCAT) . Childhood household income (from birth to age 18) were provided in 19 categories, which were subsequently summarized into five categories that approximates quintiles: <$50,000, $50,000-$74,999, $75,000-$124,999, $125,000-$199,999 and ≥$200,000. Race and ethnicity were summarized into categories, comprising White, Black/African-American, Hispanic, Native American/Alaska Native, Multiracial students who identified with more than one racial categories, and Other. Students in the Other categories included those who selected "Other" in the questionnaire.

We performed multiple imputation using a fully conditional specification method for all missing data assuming a joint distribution for all variables used for the imputation. Since imputation results were consistent with those from observed data, only results from imputed data were presented. We performed linear regression trend analyses on the distribution of students applying to and being accepted into one or more MD-PhD program by income.

© 2024 Nguyen M et al. *JAMA Network Open.*
